# Functional polymorphisms in asporin and *CILP* together with joint loading predispose to hand osteoarthritis

**DOI:** 10.1186/s12863-017-0585-4

**Published:** 2017-12-12

**Authors:** Mari Taipale, Svetlana Solovieva, Päivi Leino-Arjas, Minna Männikkö

**Affiliations:** 1 Center for Life Course Health Research, Faculty of Medicine, University of Oulu, Aapistie 5, 90220 Oulu, Finland; 2 Biocenter Oulu and Faculty of Faculty of Biochemistry and Molecular Medicine, University of Oulu, Oulu, Finland; 3 Department of Epidemiology and Biostatistics, Centre of Expertise for Health and Work Ability, Finnish Institute of Occupational Health, Helsinki, Finland

**Keywords:** Osteoarthritis, Asporin, Cartilage intermediate layer protein, Genetic association, Functional variant

## Abstract

**Background:**

Osteoarthritis (OA) is the most common degenerative joint disease afflicting people in the Western world and has a strong genetic influence. The aim of this study was to examine the association of two known functional polymorphisms in the TGF-β inhibiting genes, asporin (*ASPN*) and cartilage intermediate layer protein (*CILP*), with hand OA and potential gene-occupational hand loading interaction***.***

**Results:**

Statistically significant interaction of the *CILP* rs2073711 T and *ASPN* D15 alleles with hand OA was observed (OR = 2.48, 95% CI 1.27–4.85, *p* = 0.008) in a Finnish hand OA cohort of 543 women (aged 45–63). When stratified by variation in working tasks, low variation of working tasks increased the risk further (OR = 3.00, 95% CI 1.35–6.66, *p* = 0.007). Based on the analysis of *ASPN* and *CILP* protein-coding regions, functional studies were performed with one observed variant, rs41278695 in the *ASPN* gene. Analyses showed that bone morphogenetic protein 2 (BMP2) mediated expression of aggrecan (*Agc1)* and type II collagen (*Col2a1)* was significantly suppressed (*p* = 0.011 and *p* = 0.023, respectively) in a murine chondrocytic cell line (ATDC5) with cells stably expressing *ASPN* rs41278695.

**Conclusions:**

The carriage of either *ASPN* D15 or *CILP* rs2073711 TT is associated with increased risk of symmetrical hand OA, particularly in individuals with low variation in work tasks. *ASPN* rs41278695 SNP had an effect on *Agc1* and *Col2a1* gene expression when induced with BMP-2 suggesting an effect on the cartilage extracellular matrix composition.

## Background

Osteoarthritis (OA) is a joint disorder involving degeneration of the cartilage, but also affecting surrounding tissues [1]. OA disables often weight-bearing joints such as hip and knee, but the most frequent site is in the hand, particularly among women over 50 years of age [2]. Between 39% to 65% of the hand and knee OA in women are associated with genetic factors [3]. Despite numerous studies, only a few susceptibility genes have been identified and replicated [4]. A physiological range of mechanical loading is required for the maintenance of healthy cartilage homoeostasis [5]. Overloading as well as reduced loading increases cartilage matrix degeneration [6, 7]. Non-physiological joint loading regulates gene expression including the matrix-degrading enzymes such as the matrix metalloproteinases (MMPs) [8]. A study done with human mesenchymal stem cells demonstrated that gene regulation induced by mechanical loading is mediated through the transforming growth factor beta (TGF-β) pathway [9].

Members of the TGF-β superfamily play an important role in controlling the proliferation and differentiation of articular cartilage chondrocytes [10]. In addition, TGF-β has a crucial role in the formation of osteophytes, bony outgrowths at the margins of the joint [11]. This growth factor family includes also bone morphogenetic proteins (BMPs), activins, inhibins, and growth and differentiation factors (GDFs) [12]. Recently, Hämäläinen et al. reported a strong association between the *TGFB1* gene (transforming growth factor beta 1) and symptomatic DIP (distal interphalangeal) OA [13]. Both asporin (ASPN) and cartilage intermediate layer protein (CILP) have been shown to inhibit TGF-β mediated signaling by direct binding [14–16].

Asporin is a member of the class I subfamily of small leucine rich proteoglycans (SLRPs) although it is not a proteoglycan. It has a unique aspartic acid (D) repeat in its amino-terminal end and its expression is increased in OA [17, 18]. In addition, asporin differs from other SLRPs in relation to the ability to induce biomineralization of collagen by its calcium-binding D repeat and C-terminal collagen-binding domain [19]. ASPN D-repeat polymorphism has been associated with OA, lumbar disc degeneration (LDD) and developmental dysplasia of the hip (DDH) [14, 20, 21]. The association with OA has been replicated in small-scale studies [22–24], but several Caucasian based studies failed to replicate the association, [22, 25–27] and meta-analysis combining the data from different association studies demonstrated ethnic differences [28]. Functional studies on the *ASPN* D14 allele showed that it more greatly inhibits the TGF-β mediated expression of the aggrecan (*AGC1*) and collagen II (*COL2A1*) genes, the marker genes for chondrogenesis, than other alleles [14].

CILP is an extracellular matrix glycoprotein with a thrombospondin (TSP) type 1 repeat domain and its precursor protein encodes a homologue to porcine Nucleotide pyrophosphohydrolase (NTPPHase) in its carboxy-terminal portion. The expression of *CILP* increases with age [29, 30]. A functional single nucleotide polymorphism (SNP) in *CILP* (c.1184 T > C, rs2073711) resulting in amino acid substitution Ile395Thr is associated with lumbar disc degeneration (LDD), and the 1184C allele has a stronger inhibitory effect on TGF-β1 signaling [15]. *CILP* haplotype was also shown to associate with knee OA in men in one study [31].

Our aim was to study whether two known functional polymorphisms in the TGF-β inhibiting genes, *ASPN* and *CILP*, are associated with hand OA among women. In addition, we searched for new putative OA predisposing variants in these genes and studied in vitro the functional significance of one rare *ASPN* sequence variant that had clinical relevance. Finally, we explored the potential interaction between genetic predisposition and occupational hand loading.

## Methods

### Study population

The study material was comprised of two randomly selected samples of female dentists (*n* = 295) and teachers (*n* = 248) aged 45 to 63 years [32]. Mean age of the dentists and teachers did not differ statistically significantly: 54 years (SD 6, range 45–63) vs. 54 years (SD 4, range 45–61), respectively (*p* = 0.99). These occupational groups were chosen because of the similar socio-economic status, but different hand loading involved in daily work. The dentists have continuous and extensive manual workload that causes strain on hands and fingers, whereas the occupational description of teachers does not include straining to the joints. All individuals were living in Helsinki or its neighboring towns. They gave signed informed consent, and local ethics committee approved the study.

### Clinical and radiological assessments

Both hands of the participants were radiographed to evaluate the distal interphalangeal (DIP), proximal interphalangeal (PIP), thumb interphalangeal (IP) and metacarpophalangeal (MCP) joints of both hands by an experienced radiologist blinded to all the data regarding the subjects (occupation, age, and health data). Joints were evaluated for the presence of OA using a modified Kellgren and Lawrence (K-L) system [33] the classification criteria were: grade 0 = no OA, grade 1 = doubtful OA, grade 2 = mild OA, grade 3 = moderate OA, and grade 4 = severe OA. Participants who had symmetrical OA in at least two pairs of finger joints with radiographic OA of grade 2 to 4 were classified as having hand OA. Otherwise, the participants were classified as not having hand OA. Fouty-six randomly selected subjects were used for intraobserver and interobserver agreements by the same radiologist and by another experienced radiologist and they indicated good reliability [32]. Reference images have been reported in detail earlier [32].

### Genotyping

*ASPN* D-repeat and *CILP* rs2073711 polymorphisms were genotyped using genomic DNA extracted from lymphocytes according to standard protocols. PCR amplifications were carried out in a volume of 11–15 μl, which contained 20 ng of genomic DNA, 3 pmol of PCR primers, 1.5 mM of MgCl2, 0.2 mM dNTPs and 1 unit of Supertherm Taq DNA polymerase (Medox Biotech India) or AmpliTaq Gold DNA polymerase (Applied Biosystems). Samples were analyzed using Sanger sequencing (*CILP*) or standard fluorescence-based genotyping methodologies (*ASPN* D repeat) (ABI PRISM 3100 Genetic Analyzer, Applied Biosystems). To genotype *ASPN* D repeat, amplification products were pooled and then combined with formamide and an internal size standard (GeneScan-500, Applied Biosystems). After denaturation at 90 °C for 2 min, products were separated by size and were detected using an ABI PRISM 3100 genetic analyzer.

In addition, protein-coding regions of the *ASPN* and *CILP* genes were analyzed for rare variants by Sanger sequencing (ABI PRISM 3100 Genetic Analyzer and BigDye terminator cycle sequencing chemistry, Applied Biosystems) in the whole study population.

### Statistical analysis

Fisher’s exact probability test or the chi-square test were used to compare allele and genotype frequencies between dentists and teachers. Fisher’s exact test was chosen when the observed values were small in the individual groups, especially when comparing allele frequencies in the contingency tables. The association between the *ASPN* repeat polymorphism and *CILP* rs2073711 genotypes (CC, CT and TT) with hand OA was studied with a set of logistic regression analyses. The dependent variable was symmetrical OA of grade 2 or more in at least two joint pairs and the independent variables were *ASPN* and *CILP* genotypes. Prevalence of hand OA was statistically significantly higher among teachers than among dentists (24.7% vs. 17.0%, *p* = 0.03) and therefore age and occupation were included in the model as potential confounders. The association of polymorphisms with hand OA was also examined for dentists and teachers separately. A *p*-value of <0.05 was considered as significant. The analyses were performed with the Statistical Package for the Social Sciences, version 12.0.1 (SPSS Inc., Chicago, IL, USA).

### ASPN constructs

*ASPN* cDNA was amplified using Human Ovary Marathon-Ready cDNA (Clontech) as a template. PCR primers included recognition sequences for BamHI and XhoI restriction enzymes (primer sequences are available on request). The amplified PCR product was ligated into pcDNA3.1(+) expression vector (Invitrogen). The mutant *ASPN* construct was generated using QuikChange Site-Directed Mutagenesis Kit (Stratagene) according to the manufacturer’s instructions. Primer sequences used in mutagenesis are available on request.

### Cell culture and transfections

A murine chondrocytic cell line (ATDC5) was purchased from RIKEN Cell Bank, Tsukuba Japan. ATDC5 cells were cultured in 1:1 mixture of Dulbecco’s modified Eagle’s medium (DMEM; Biochrom KG, Berlin, Germany) and Ham’s F-12 nutrient mixture supplemented with 5% (*v*/v) fetal bovine serum (FBS; GB Perbio HyClone, Cheshire, UK), 10 μg/ml human transferrin (Sigma), 3 × 10^−8^ M sodium selenite (Sigma), 0.1% penicillin (Sigma, St Louis, MO), 0.01% Fungizone (Cambrex Bio Science, Walkersville, MD), 0.1% L-glutamate (Sigma) and 5% Na-bicarbonate (Sigma) at 37 °C under 5% CO_2_.

For transient transfections 3.5 × 10^5^ cells were plated on 6-well plates and cultured in maintenance medium with ITS (Sigma; 10 μg/ml insulin, 10 μg/ml transferrin and 3 × 10^−8^ M sodium selenite). After 24 h cells were transfected with *ASPN* wild type (wt) or mutant construct using Lipofetamine and Plus-reagent (Invitrogen) according to manufacturer’s instructions. Empty pcDNA3.1 (+) was used as a negative control, and 2 μg DNA, 3 μl Lipofectamine and 10 μl Plus-reagent were used. Forty-eight hours after transfections medium was changed to DMEM-F12 (1:1) supplemented with 0.2% FBS and ITS. Twelve hours after the medium change cells were treated with 10 ng/ml TGF-β1 (Chemicon) for 16 h or six hours after medium change with 100 ng/ml BMP-2 (ProSpec) for 12 h. Each experiment was performed in sextuple and repeated three times.

For generating stable cell line expressing wt or mutant *ASPN* 3.5 × 10^5^ cells were plated on a 6-well plate and cultured in the maintenance medium for 24 h. Cells were transfected with *ASPN* wt or mutation construct using Lipofetamine and Plus-reagent as described earlier. Empty pcDNA3.1 (+) was used as a negative control. After 24 h the medium was replaced with fresh maintenance medium supplemented with 500 μg/ml Geneticin, G418 (Sigma). The selection medium was changed every second day until cells without the neomycin resistance gene on the control plate were killed. After 11d, cells were diluted and plated on a 96-well plate. Selection of single colony cells was done using serial dilution.

Clones stably expressing wt or mutant *ASPN*, and a clone with an empty pcDNA3.1 (+) vector were cultured in maintenance medium with 500 μg/ml G418 until they reached confluence. After that 3.5 × 10^5^ cells were plated on a 6-well plate. After 24 h the standard medium was supplemented with ITS and 50 μg/ml ascorbic acid. After 24 h the cells were treated with 10 ng/ml TGF-β1, 100 ng/ml BMP-2 or without any growth factor for 24 h. Each experiment was performed in sextuple and two independent clones for each asporin variant were used.

### Real-time quantitative polymerase chain reaction (qPCR)

Total RNA was extracted from cells using E.Z.N.A Total RNA Kit (Omega Bio-Tek) with RNase-free DNase (Omega Bio-Tek) treatment and cDNA was synthesized using iScript cDNA Synthesis Kit (Bio-Rad). Real-time qPCR was carried out in duplicate using iTaq SYBR Green Supermix with ROX kit (Bio-Rad) in accordance with the manufacturer’s instructions in an MXPro 3005 machine (Stratagene). *Gapdh* (glyceraldehyde 3-phosphate dehydrogenase) was used as the reference gene. Sequences of oligonucleotide primers used in real-time qPCR amplification are available on request.

## Results

### Association of ASPN D repeat and CILP rs207371

The *ASPN* and *CILP* variants were analyzed in a sample of Finnish women from two occupational groups (295 dentists and 248 teachers). Genotyping of both variants were successful from 524 women. The allelic frequencies of the *ASPN* D repeat and *CILP* rs2073711 polymorphisms are summarized in Table 1. The *ASPN* D15 allele was overrepresented in the teachers as compared with the dentists (Fisher’s exact probability test, *p* = 0.007) and therefore stratification of the data by occupation was used in the analyses. Dentists carrying at least one *ASPN* D15 allele or *CILP* rs2073711 TT had an increased risk for hand OA (OR = 2.48, 95% CI 1.27–4.85, *p* = 0.008) compared to teachers (Table 2). When stratified by variation in dental tasks, low variation of dental tasks increased the risk further (OR = 3.00, 95% CI 1.35–6.66, *p* = 0.007) (Table 2). There was a synergetic effect of joint overuse and having either *ASPN* D15 or *CILP* rs2073711 TT on the risk of symmetrical hand OA when analyzing dentists only (OR = 3.30, 95% CI 1.18–9.20, *p* = 0.02) (Table 3).

**Table 1:** Allelic frequencies of the *ASPN* D repeat and *CILP* rs2073711 polymorphisms

Alleles	All (*N* = 524)		Dentists (*N* = 285)		Teachers (*N* = 239)	
	N (cases)	%	N (cases)	%	N (cases)	%
*ASPN* D repeat						
D12	23 (2)	2.2	14 (1)	2.5	9 (1)	1.9
D13	555 (114)	53.0	310 (45)	54.4	245 (69)	51.3
D14	172 (35)	16.4	101 (20)	17.7	71 (15)	14.9
D15	190 (46)	18.1	86 (21)	15.1	104 (25)	21.8
D16	65 (11)	6.2	33 (4)	5.8	32 (7)	6.7
D17	26 (4)	2.5	18 (3)	3.2	8 (1)	1.7
D18	17 (2)	1.6	8 (2)	1.4	9 (0)	1.9
*CILP* rs2073711	All (*N* = 529)		Dentists (*N* = 288)		Teachers (*N* = 241)	
	N (cases)	%	N (cases)	%	N (cases)	%
T	435 (92)	41.1	233 (42)	40.4	202 (50)	41.9
C	623 (124)	58.9	343 (54)	59.6	280 (70)	58.1
CC	180 (33)	34.0	106 (17)	36.6	74 (16)	30.7
CT	263 (58)	48.4	131 (20)	45.5	132 (38)	54.8
TT	86 (17)	16.3	51 (11)	17.7	35 (6)	14.5

Fisher’s exact probability test was used to compare allele frequencies between dentists and teachers and a chi-square test was used to compare genotype frequencies between dentists and teachers

**Table 2:** Association of *ASPN* D repeat and *CILP* rs2073711 polymorphism with hand OA

*ASPN* D15 & *CILP* rs2073711	N (cases^a^)	OR (95% CI)^b^	Stratified by occupation Dentists / Teachers OR (95% CI)	Stratified by variation in dental tasks (dentists only) - Low variation OR (95% CI)
No D15 & CC + CT	300 (54)	1.0	1.0	1.0
At least one D15 or TT	224 (53)	1.43 (0.92–2-23)	2.48 (1.27–4.85), *p* = 0.008^c^/ 0.91 (0.50–1.67), *p* = 0.77	3.00 (1.35–6.66), *p* = 0.007^d^

Logistic regression analysis; CI, confidence interval; OR, odds ratio

^a^Symmetrical OA of grade 2 or more in at least two joints pairs

^b^Adjusted for age (years) and occupation

^c^Bonferroni corrected *p* = 0.04

^d^Bonferroni corrected *p* = 0.035

**Table 3:** Joint effect of *ASPN* D15 and *CILP* TT variations, and variation in dental task among dentists in relation to hand OA

*ASPN* D15 & *CILP* rs2073711	Variation in dental tasks	Symmetrical OA in at least two joint pairs		
		N (cases)	OR (95% CI)^a^	*p*-value
No D15 & CC + CT	diversity of tasks	58 (6)	1.0	
No D15 & CC + CT	low variation	109 (15)	1.14 (0.40–3.25)	0.80
At least one D15 or TT	diversity of tasks	35 (5)	1.91 (0.50–7.25)	0.34
At least one D15 or TT	low variation	77 (22)	3.30 (1.18–9.20)	0.02

Logistic regression analysis, CI, confidence interval; OR, odds ratio

^a^Adjusted for age (years)

### Rare coding variants

Coding sequences and exon-intron boundaries of the *ASPN* and *CILP* genes were analyzed in the whole study population to find rare variants. A missense SNP causing a Gly193Glu change in the fourth exon of *ASPN* was found in ten individuals (1.8%). Because of the low frequency, our sample set was not sufficient for association analyses. The clinical status of the variant carriers was evaluated more closely. Seven of the ten individuals had osteophytes or at least grade 2+ OA in at least one finger joint. The remaining three individuals did not have OA or any chronic diseases diagnosed by the physician, but at the time of the enrollment they all had reported pain in the joints of the fingers during the past 30 days, two had reported wrist pain during the past 12 months and one had shoulder pain, one neck pain and two had low back pain during the past 12 months. This variant (rs41278695) has been annotated in the 1000 genome project with minor allele frequency of less than 0.01. Tomoeda et al. has showed that rs41278695 inhibits BMP-2 signaling in periodontal ligament (PDL) cells [16]. Based on the patients’ status and suggestion on its functionality, rs41278695 was chosen for functional assays.

### Functional significance of rs41278695

**Fig. 1:**
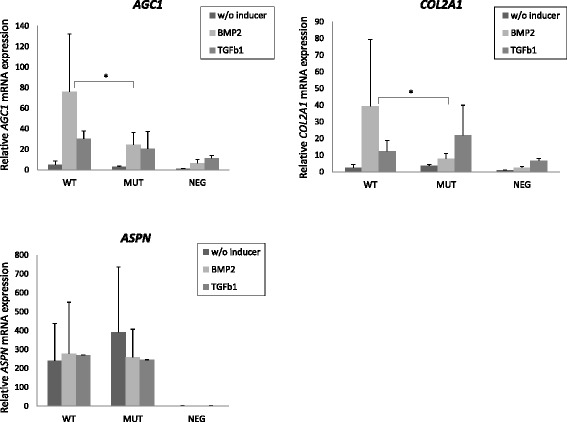
Effect of the stable overexpression of wt ASPN (WT) and ASPN-193Glu (MUT) on the expression of cartilage marker genes during chondrogenic differentiation of ATDC5 cells. Error bars represent standard deviation of six biological replicates of two independent clones

The effect of *ASPN* rs41278695 on the TGF-β1 mediated expression of aggrecan and collagen II was studied. Expression levels of *Agc1* and *Col2a1* was measured in ATDC5 cells transfected with wt or mutant *ASPN* construct and treated with TGF-β1 or BMP2. Expression of *Agc1* and *Col2a1* was not changed between ATDC5 cells transiently transfected with wt *ASPN* or mutant *ASPN* expressing plasmids (data not shown). Also, there was no difference when TGF-β1 or BMP2 was used as an inducer. However, *Agc1* and *Col2a1* expression was suppressed (*p* = 0.011 and *p* = 0.023, respectively) in ATDC5 cells stably expressing mutant *ASPN* compared to cells expressing wt *ASPN* (Fig. 1). This was noticed only when BMP2 was used as inducer. There was no difference in the *ASPN* expression between the clones.

## Discussion

In the present study, we demonstrate that there is a synergetic effect of joint overuse and genetic variants on the risk of symmetrical hand OA. Dentists having either *ASPN* D15 or *CILP* rs2073711 TT and having low variation of dental tasks had increased risk for hand OA. Our study also supports previous findings that ASPN is a regulator of BMP-2 mediated activation of chondrogenesis.

In previous studies, *ASPN* D14, instead of D15, was associated with knee OA, especially in studies performed in Asian populations [14, 23, 24]. However, a Greek study demonstrated an association between D15 allele and knee OA [26], which is consistent with our finding. Contrary to the study of Kizawa et al., the protective role of *ASPN* D13 was not seen in the present study. To the best of our knowledge, this is the first study that shows the association between ASPN D-repeat and hand OA.

Our findings suggest that *CILP* rs2073711 TT is associated with increased risk of symmetrical hand OA together with *ASPN* D15 allele. The *CILP* rs2073711 TT genotype was associated with disc degeneration (DD) in a Finnish study [34]. In addition, *CILP* rs2073711 genotype CC was shown to associate with lower radiographic progression of OA in a UK study [35]. However, contradictory results were obtained in a Japanese case-control study, which showed an association between the *CILP* rs2073711 C allele and LDD [15]. Seki et al. also demonstrated that C allele of *CILP* rs2073711 showed increased binding and inhibition of TGF-β signaling. It has been indicated in several association studies that there are ethnic differences between Asian and Caucasian populations [28, 36–40]. For instance, allele frequency of *CILP* rs2073711 C is more common in Europeans than in Asians (The NCBI dbSNP database) and both alleles of certain polymorphisms have been associated with osteoporosis and gastric cancer among different racial groups [41–43]. One explanation is that the alleles are in a different haplotype blocks in different populations and the other factors define if the allele has a harmful or protective role in a population [44].

While physiological range of mechanical loading maintains cartilage homeostasis, both reduced loading and overloading shift the metabolic reactions to favor catabolism over anabolism [6]. It has been shown that low variation in work tasks, for example in dental tasks, is a risk factor for finger OA [45]. We observed the same phenomenon in the present study. When the results were stratified by variation in dental tasks, the risk for finger OA was increased in women having either *ASPN* D15 or *CILP* rs2073711 TT genotype and low variation in joint usage. Hämäläinen et al. used the same hand OA cohort in their study and showed that *COL2A1* gene polymorphisms may play a role in the aetiology of hand OA together with repetitive loading work tasks [46]. One limitation of our study was in its modest sample size for a genetic study of a complex disease. It has however the advantage that the study subjects were well characterized for their OA status and the use of hand joints. Our results presented here further supports the hypothesis that mechanical stress such as joint loading has an effect on the expression of cartilage matrix genes [47] and thus regulates the gene expression together with genetic variations.

In addition, we identified a rare missense SNP rs41278695 resulting in an amino acid change Gly193Glu in the human *ASPN* gene among hand OA patients with osteophytes. This sequence variation is located at the fifth leucine rich repeat (LRR5) of asporin. Asporin binds directly to TGF-β and binding is mediated through amino acids 159–205 [48]. This sequence also contributes the induction of collagen II and aggrecan gene expression [48]. According to our results, Gly193Glu had an effect on *Agc1* and *Col2a1* gene expression when induced with BMP-2. This effect was not shown when we used TGF-β1 as an inducer. There was relative high variation in the measurements of the biological replicates especially for the wt, but our finding is consistent with an earlier study by Tomoeda et al., which showed that asporin inhibits BMP-2 signaling via LRR5 and Glu-194 (the amino acid numbering depends on the D repeat length) plays critical role in the interaction [16]. As well as asporin other genes which belong to TGF-β signaling pathway are associated with osteoarthritis, including *GFD5* [49–53] and *SMAD3* [54, 55].

Our study suggests that combinations of variants in the *ASPN* and *CILP* gene, which both act in the TGF-β pathway, possibly have an additive effect in the development of hand OA. The combinatorial effect between *ASPN* and *CILP* suggests a cross-talk between these two genes. This effect is enhanced by high loading of joints. This is the first study that shows association between *ASPN* D repeat and *CILP* rs2073711 polymorphisms and hand OA. The in vitro studies of the Gly193Glu amino acid change further supports the important role of rare variants in common diseases such as osteoarthritis. In complex diseases, like in osteoarthritis, several loci have been associated with the disease in large-scale genome-wide association studies (GWAS) [56, 57]. Common SNPs have only small effect sizes and thus are not likely to play a major role in development of the disease. Rare variants, however, account for a greater faction of familial clustering of common diseases, and have a strong impact on the disease risk [58]. Taken together variants in the TGF-β signaling pathway molecules seem to play a major role in the development of OA and joint loading enhances the effect. Thus modulation of TGF-β signaling pathway may be a potential therapeutic target for osteoarthritis.

## Conclusions

The present study provides further evidence for the role of *ASPN* and *CILP* polymorphisms in the etiology of OA. This effect was further increased by mechanical hand loading supporting the hypothesis of gene - environmental interaction as part of the etiology in hand OA.

## Abbreviations

ASPN: Asporin
BMP: Bone morphogenetic protein
CI: Confidence interval
CILP: Cartilage intermediate layer protein
D: Aspartic acid
DDH: Developmental dysplasia of the hip
DIP: Distal interphalangeal
DMEM: Dulbecco’s modified Eagle’s medium
FBS: Fetal bovine serum
Gapdh: Glyceraldehyde 3-phosphate dehydrogenase
GDF: Differentiation factor
Glu: Glutamic acid
Gly: Glycine
IP: Thumb interphalangeal
ITS: Insulin-transferrin-sodium selenite
K-L: Kellgren and Lawrence
LDD: Lumbar disc degeneration
MCP: Metacarpophalangeal
MMP: Matrix metalloproteinase
NTPPHase: Nucleotide pyrophosphohydrolase
OA: Osteoarthritis
OR: Odds ratio
p: *P*-value
PIP: Proximal interphalangeal
SLRP: Small leucine rich proteoglycan
SNP: Small nucleotide polymorphism
TGF-β: Transforming growth factor beta
TSP: Thrombospondin
WT: Wild type
